# Preemptive Administration of Albumin during Pancreatectomy Does Not Reduce Postoperative Complications: A Prospective Randomized Controlled Trial

**DOI:** 10.3390/jcm11030620

**Published:** 2022-01-26

**Authors:** Heejoon Jeong, Jie Ae Kim, Mikyung Yang, Hyun Joo Ahn, JinSeok Heo, In Woong Han, Sang Hyun Shin, Nam Young Lee, Woo Jin Kim

**Affiliations:** 1 Department of Anesthesiology and Pain Medicine, Samsung Medical Center, Sungkyunkwan University School of Medicine, Seoul 06351, Korea; heejoon.jeong@samsung.com (H.J.); anes.yang@samsung.com (M.Y.); hyunjooahn@skku.edu (H.J.A.); namyoung2.lee@samsung.com (N.Y.L.); wj888.kim@samsung.com (W.J.K.); 2 Department of Surgery, Samsung Medical Center, Sungkyunkwan University School of Medicine, Seoul 06351, Korea; jinseok.heo@samsung.com (J.H.); iw.han@samsung.com (I.W.H.); sanghyun80.shin@samsung.com (S.H.S.)

**Keywords:** preemptive albumin, pancreatectomy, postoperative complication

## Abstract

Despite the empirical use of human albumin during pancreatectomy to replace intraoperative volume loss while preventing fluid overload and edema, its impact on postoperative outcomes remains unclear. In addition, most previous studies have focused on the effects of therapeutic albumin usage. Here, we investigated whether preemptive administration of human albumin to prevent edema during pancreatectomy could reduce the incidence of moderate postoperative complications. Adult patients undergoing pancreatectomy were assigned to either the albumin group (*n* = 100) or the control group (*n* = 100). Regardless of the preoperative albumin level, 200 mL of 20% albumin was administered to the albumin group after induction of anesthesia. The primary outcome was the incidence of moderate postoperative complications as defined by a Clavien–Dindo classification grade ≥ 2 at discharge. Intraoperative net-fluid balance, a known risk factor of postoperative complication after pancreatectomy, was lower in the albumin group than in the control group (*p* = 0.030), but the incidence of moderate postoperative complications was not different between the albumin and control groups (47/100 vs. 38/100, respectively; risk ratio: 1.24, 95% CI: 0.89 to 1.71; *p* = 0.198). Therefore, preemptive administration of human albumin to prevent fluid overload and edema during pancreatectomy is not recommended because of its lack of apparent benefit in improving postoperative outcomes.

## 1. Introduction

Pancreatic resection is one of the most aggressive abdominal surgeries, and many strategies to enhance postoperative outcomes have been investigated. Most pancreatic resections take a longer operative time, require many anastomoses, and lead to a fluid shift due to an inflammatory reaction. In addition, perioperative high net-fluid balance is known as a risk factor for pancreatic fistula after pancreaticoduodenectomy (PD) [1,2]. Because tissue or bowel edema due to excessive fluid infusion during pancreatectomy could interfere with surgical anastomosis and result in postoperative complications, many surgeons and anesthesiologists have reduced the volume of crystalloid used, and alternatively used colloids such as human albumin to maintain intravascular volume while preventing fluid overload and edema. However, the impact of preemptive albumin treatment to prevent the occurrence of fluid overload and edema on postoperative outcomes remains unclear.

Recently, multiple studies have suggested a relevant association between perioperative hypoalbuminemia and poor postoperative surgical outcomes [3,4,5] and investigated the effect of albumin replacement in these surgical patients [6,7]. However, most previous studies have focused on the effect of the therapeutic use of albumin in patients who were already diagnosed with perioperative hypoalbuminemia. To the best of our knowledge, no studies have assessed the effect of the preemptive use of albumin to prevent fluid overload and edema on postoperative outcomes in surgical patients. Because serum albumin level usually reduces after major abdominal surgeries, preemptive infusion of albumin can mitigate the decrease in serum albumin level and alleviate the influence of postoperative hypoalbuminemia.

Although therapeutic albumin replacement after PD did not improve postoperative prognosis in a previous study [8], this is not sufficient evidence against preemptive or prophylactic administration of albumin to prevent fluid overload and edema during pancreatectomy. Caironi et al. [9] reported that albumin replacement contributed to maintaining mean arterial pressure and decreasing the net-fluid balance in patients with severe sepsis. If preemptive albumin treatment reduces the net-fluid balance and prevents edema during pancreatectomy, it can also lead to decreased postoperative complication rates.

Therefore, we investigated the effect of preemptive and empirical albumin administration to prevent fluid overload and edema on postoperative outcomes in patients undergoing pancreatectomy. We hypothesized that preemptive administration of albumin would reduce the incidence of moderate postoperative complications by lowering the net-fluid balance during pancreatectomy. Our primary endpoint was the incidence of Clavien–Dindo classification grade ≥ 2 at discharge after pancreatectomy.

## 2. Materials and Methods

### 2.1. Study Design

This prospective, randomized, and controlled study was conducted at a tertiary academic hospital in Seoul, Korea. The study protocol was approved by the Institutional Review Board of the Samsung Medical Center, Seoul, Korea (approval number: 2017-08-096-002; approval date: 17 October 2017) and registered with the Korean Clinical Research Information Service (registration number: KCT0002759; principal investigator: Jie Ae Kim; date of registration: 28 March 2018; accessed on: 2 June 2021; https://cris.nih.go.kr). Our study was conducted in accordance with the ethical principles of the 1964 Declaration of Helsinki and its later amendments. Written informed consent was obtained from all participants. The manuscript adheres to the applicable consolidated standards of reporting trials (CONSORT) guideline.

### 2.2. Participants

Between April 2018 and October 2019, 205 patients were scheduled for elective pancreatectomy (i.e., pylorus-preserving or resecting PD, Whipple’s operation, radical antegrade modular pancreatosplenectomy (RAMPS), and total pancreatectomy) and were assessed for eligibility and contacted by primary investigators a day before surgery to obtain written informed consent.

Patients aged between 20 and 80 years with American Society of Anesthesiologist physical status I to III were included. Exclusion criteria were as follows: cardiac ejection fraction < 40%, myocardial infarction history within 3 months, renal dysfunction (serum creatinine > 1.8 mg/dL), platelet count < 100,000/µL, international normalized ratio > 1.5, body mass index ≥ 35 kg/m^2^, active sepsis or bacteremia, uncontrolled diabetes mellitus (Hemoglobin A1C > 7.0% or fasting serum glucose > 200 mg/dL), and the use of prednisolone (>10 mg/day) or its equivalent. Dropout criteria included withdrawal of consent, cancellation of surgery before randomization, and unstable hemodynamics during induction of anesthesia, which defined vital signs that could not be maintained within the target range (20% of baseline values measured before induction of anesthesia) despite administration of fluid or inotropes/vasopressors. According to the intention-to-treat principle, all randomized participants were included in the analysis.

### 2.3. Randomization and Blinding Method

Patients, surgeons, and anesthesiologists were blinded to the group assignments. Randomization was performed using a computer-generated random numbers table with a fixed block size of four and a 1:1 ratio. Allocation was sequentially numbered and sealed in opaque envelopes by the corresponding author. After induction of anesthesia, the study staff opened the envelope, prepared the study fluid according to allocation, and administered it to the patient.

### 2.4. Interventions

Two anesthesiologists performed each case. The first anesthesiologist induced general anesthesia and conducted the intervention assigned to the patient. A total of 200 mL of 20% human albumin (Green Cross Co., Gyeonggi, Korea) was administered to the albumin group. The same volume of balanced crystalloid (Plasma solution A^®^, HK inno.N Corp., Seoul, Korea) was administered to the control group. All the study fluids were infused at a rate of 4 mL/kg/hr. After infusion of the study fluid, the attending staff was switched to a second anesthesiologist who was blinded to the group assignment.

### 2.5. Anesthesia and Fluid Management

The induction and maintenance of anesthesia were standardized and identical for all patients. General anesthesia was initiated with intravenous propofol (1.5–2 mg/kg) and rocuronium (0.8 mg/kg), and maintenance was achieved using a 1.0 minimum alveolar concentration of sevoflurane and continuous remifentanil infusion (0.05–0.20 µg/kg/min) within a bispectral index of 45 ± 5. Arterial cannulation for invasive hemodynamic monitoring was performed, and cardiac output and stroke volume variation (SVV) were continuously monitored using the FloTrac^TM^ system connected to an EV-1000 platform (Edwards Lifesciences, Irvine, CA, USA). The target of anesthetic management was to maintain the mean arterial pressure within 20% of the baseline values. Phenylephrine, ephedrine, or nicardipine was administered as required to maintain values within this range. If the heart rate was less than 40 beats/min, 0.5 mg of atropine was administered.

A balanced crystalloid (Plasma solution A^®^) was used as the maintenance fluid and given at a rate of 4 mL/kg/h. Intraoperative fluid management was conducted using goal-directed fluid therapy (GDFT) based on SVV. An additional bolus of 200 mL crystalloid was administered for 15 min when the SVV was ≥15%, and this was repeated every 30 min. If mean arterial pressure was maintained over 65 mmHg or urine output was measured at a rate of 0.5 mL/kg/h or more, a bolus of crystalloid was not administered to prevent fluid overload. When the mean arterial pressure decreased below the target value despite optimizing the circulating volume, ephedrine or phenylephrine administration was considered. A transfusion was performed for effective resuscitation in patients with hemoglobin levels below 8 g/dL.

All patients received intravenous hydromorphone (0.01 mg/kg) 30 min before the end of surgery. The neuromuscular blockade was reversed with intravenous pyridostigmine (0.2 mg/kg) and glycopyrrolate (0.008 mg/kg). Most patients were extubated when they recovered adequate muscle strength and were transferred to the post-anesthesia care unit (PACU) or intensive care unit (ICU).

### 2.6. Surgical Techniques and Postoperative Management

Three experienced surgeons who were blinded to the group allocation performed all the surgical procedures and postoperative care according to the uniform and standardized protocol at our institute. Retrocolic hepaticojejunostomy, pancreaticojejunostomy, and antecolic duodenojejunostomy were used as standard reconstruction methods for PD. The duct-to-mucosa method was performed for most pancreaticojejunostomy reconstruction procedures. During RAMPS, the hand-sewn method was used for surgical stump closure. Portal dissection included clearance of the portal vein, hepatic artery lymph nodes, and celiac lymphadenectomy.

After surgery, patients who underwent PD were transferred to the ICU directly and closely monitored until postoperative day (POD) 1. In the case of RAMPS, most patients were transferred to the PACU, and then to the general ward.

If patients showed serum albumin < 3.0 g/dL in the postoperative period, 100 mL of 20% human albumin (Green Cross Co., Gyeonggi, Korea) was administered according to the discretion of the surgeons. Transfusion was performed in patients who reported anemia (hemoglobin 8.0 < g/dL) or thrombocytopenia (platelet count < 50,000/µL).

### 2.7. Study Outcomes and Measurements

The primary outcome was the incidence of moderate postoperative complications defined by a Clavien–Dindo classification grade ≥ 2 after pancreatectomy at the first discharge. The secondary endpoints included the net-fluid balance, incidence of intraoperative oliguria, intraoperative cardiac index, use of synthetic colloids, use of inotrope/vasopressor, intraoperative blood loss, intra- and postoperative transfusion, and changes in serum albumin levels until POD 5. In patients who underwent PD, predictive scores such as the pancreatic fistula risk score [10,11] and Braga score [12] were calculated (the definitions of each scoring system are presented in Table S1). The Clavien–Dindo classification grade, clinically relevant postoperative pancreatic fistula (CR-POPF) grade (if available), and other major postoperative complications were recorded. CR-POPF was defined according to the International Study Group for Pancreatic Fistula Classification (ISGPF) [13]. Other major complications were defined according to the International Study Group of Pancreatic Surgery (ISGPS). All perioperative variables were measured and recorded by the study staff, who were blinded to the group allocation.

### 2.8. Statistical Analysis

The incidence of Clavien–Dindo classification grade ≥ 2 after pancreatectomy was approximately 48% in our institute. We hypothesized that preemptive administration of albumin to prevent fluid overload and edema in patients undergoing pancreatectomy could reduce the incidence of moderate postoperative complications by 40%. Thus, we expected the incidence of postoperative complications to be 28.8% in the albumin group. With a two-tailed significance level of 0.05 and a power of 80%, 200 study subjects were required. All cases were analyzed using the intention-to-treat principle.

Continuous variables were presented as means ± standard deviations (SDs) or medians (interquartile range). Categorical variables are presented as numbers (%). The normal distribution of the data was evaluated using the Shapiro–Wilk test. Confidence intervals (CIs) for non-normally distributed variables were calculated using the Hodges–Lehmann estimator. The differences in continuous variables between the albumin and control groups were compared using an independent *t*-test or Mann–Whitney U-test, depending on the data distribution. A chi-square test or Fisher’s exact test was used for categorical variables, and absolute risk difference and effect size were evaluated by computing the risk difference and risk ratio with 95% CIs for a binary outcome.

Standardized mean differences (SMDs) of patient characteristics and intraoperative variables were calculated to identify possible imbalances between the groups. Multiple logistic regression analysis was performed to identify the predictors of Clavien–Dindo classification grade ≥ 2 after pancreatectomy using all variables that showed an SMD > 0.1 between the groups in the baseline analysis. We used a logistic regression model to adjust for major confounders, such as type of surgery and preoperative biliary drainage. The change in serum albumin level after pancreatectomy was analyzed using repeated-measures analysis of variance (ANOVA) and adjusted using Bonferroni correction. Subgroup analysis of patients who underwent PD was performed.

All *p*-values were two-sided, and *p* < 0.05 was considered significant. All data were analyzed using MedCalc 14.12.0 (MedCalc; MedCalc Software Co. Ltd., Ostend, Belgium).

## 3. Results

**Figure 1:**
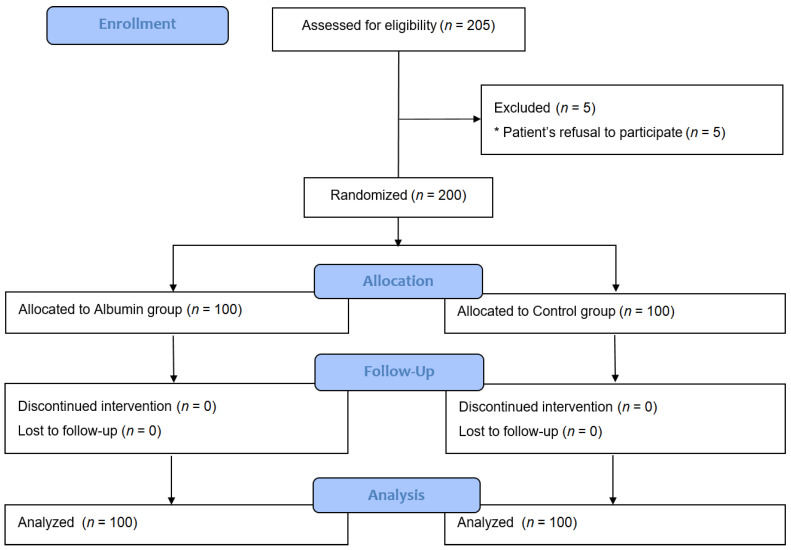
Consolidated standards of reporting trials flow diagram of the study.

A total of 205 patients were assessed for eligibility. However, five patients were excluded because they refused to participate. The remaining 200 patients were randomized and received their allocated treatment. All participants completed the study and were analyzed (Figure 1). There were no adverse events related to the study protocol in either group.

The baseline characteristics of the participants, diseases, surgeries, and anesthesia of the two groups are presented in Table 1. The incidence of preoperative biliary drainage was 30% and 45% in the control and albumin groups, respectively (SMD = 0.314; *p* = 0.041).

**Figure 2:**
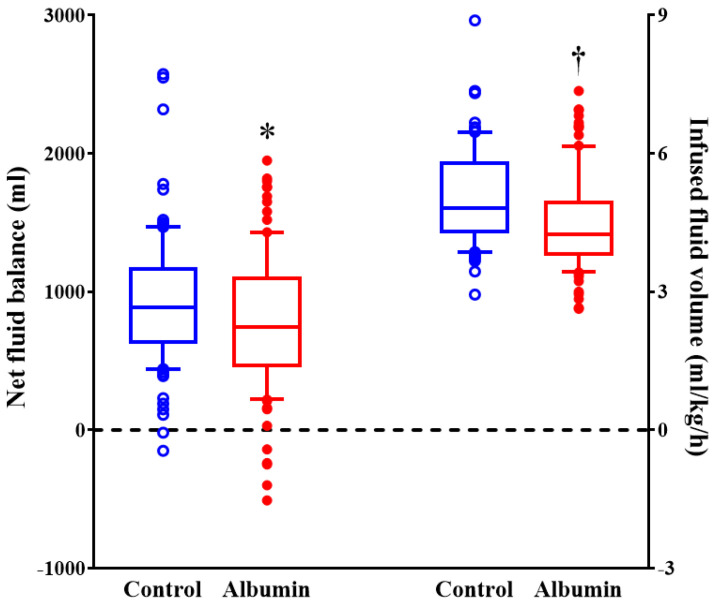
The volume of intraoperative fluid during pancreatectomy. Horizontal lines, boxes, and error bars represent the median, interquartile range, and outliers, respectively. * *p* = 0.03 versus control group. † *p* < 0.001 versus control group.

The volume of intraoperative crystalloid considering body weight and operation duration was 4.8 mL (4.3–5.8) and 4.3 mL (3.8–5.0), respectively (per kg/h; median (interquartile range); *p* = 0.001), and net-fluid balance was 920 ± 471 mL and 770 ± 495 mL, respectively (mean ± SD; *p* = 0.030) (Figure 2).

**Figure 3:**
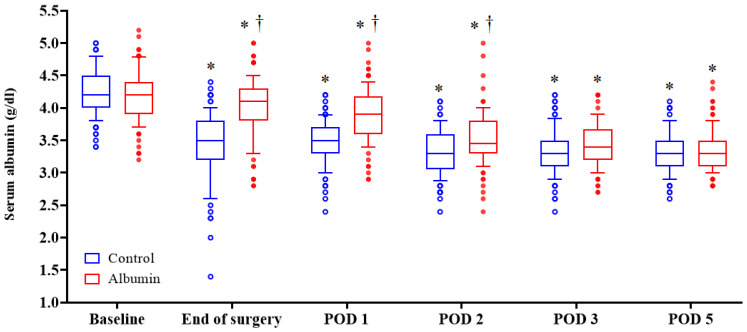
Postoperative changes of serum albumin levels in both groups. Horizontal lines, boxes, and error bars represent the median, interquartile range, and outliers, respectively. * *p* < 0.05 versus baseline value. † *p* < 0.05 versus control group. All *p* values were adjusted by Bonferroni correction. POD: postoperative day.

The serum albumin level decreased after pancreatectomy in both the groups. The median serum albumin level in the control group was significantly lower than that in the albumin group at the end of the surgery, POD 1, and POD2 (*p* < 0.001, *p* < 0.001, *p* = 0.006, respectively; *p* values were adjusted by Bonferroni correction) (Figure 3).

The incidence of Clavien–Dindo classification grade ≥ 2 at discharge was 38% and 47% in the control and albumin groups, respectively (risk ratio: 1.24, 95% CI: 0.89 to 1.71; *p* = 0.198) (Table 2).

In multiple logistic regression analysis, intraoperative albumin administration was not included in the predictors of Clavien–Dindo classification grade ≥ 2 after pancreatectomy (odds ratio: 1.61, 95% CI: 0.76 to 3.40; *p* = 0.215), while preoperative biliary drainage (odds ratio: 3.63, 95% CI: 1.94 to 6.80; *p* < 0.001) and a longer duration of surgery (per min; odds ratio: 1.01, 95% CI: 1.00 to 1.01; *p* = 0.002) increased the risk of moderate postoperative complications (Table 3).

Even after adjusting for major confounders, including preoperative biliary drainage and type of surgery using a logistic regression model, preemptive albumin infusion did not reduce the incidence of a Clavien–Dindo classification grade ≥ 2, but a longer duration of surgery remained a relevant risk factor for Clavien–Dindo classification grade ≥ 2 after pancreatectomy (per min; odds ratio: 1.01, 95% CI: 1.00 to 1.01; *p* = 0.015).

In the subgroup analysis of patients who underwent PD, which made up the majority of participants, and who underwent the most invasive procedure in this study, the incidence of a Clavien–Dindo classification grade ≥ 2 at discharge was 43% and 52% in the control and albumin groups, respectively (risk ratio: 1.19, 95% CI: 0.86 to 1.65; *p* = 0.290) (Table S2).

## 4. Discussion

In the current study, preemptive administration of human albumin in patients undergoing pancreatectomy reduced the net-fluid balance but did not reduce the incidence of Clavien–Dindo classification grade ≥ 2. In addition, a longer duration of surgery and preoperative biliary drainage were relevant risk factors for a Clavien–Dindo classification grade ≥ 2.

Albumin is a major protein making up human plasma and is essential for maintaining plasma oncotic pressure, which accounts for approximately 80% of the total oncotic pressure [14]. Therefore, a reduction in serum albumin concentration is an important determinant of the appearance of a patient’s edema. During surgery, extravascular loss of albumin derived from endothelial glycocalyx (EG) layer injury [15] and dilution of serum albumin concentration by fluid therapy can result in edema. In fact, edema due to immoderate fluid management during PD was associated with the development of postoperative complications in a previous study [16]. Moreover, Weinberg et al. [2] reported that restrictive fluid therapy and negative cumulative fluid balance contributed to a lower rate of complications and a shorter duration of hospital stay after PD. In these aspects, exogenous albumin has been empirically used to replace intraoperative volume loss while minimizing edema in patients undergoing PD, even in patients without significant hypoalbuminemia. However, there is no randomized trial on the impact of albumin replacement prior to the decrease in albumin level on postoperative outcomes.

Recently, perioperative serum albumin level has been regarded as a feasible predictor of postoperative outcomes in patients with malignancy [17]. Cancer-induced higher metabolism, impaired protein synthesis, and decreased dietary intake due to anorexia or dyspepsia are responsible for preoperative hypoalbuminemia in cancer patients [18]. These impairments usually continue throughout the postoperative period and cause delayed recovery or postoperative complications. Moreover, preoperative hypoalbuminemia is a known risk factor for pancreatic fistula and other complications, including postoperative bleeding and multiple organ failure after PD [19]. Therefore, some clinicians aim to maintain serum albumin levels within a normal range during the perioperative period using exogenous albumin. However, the benefits and exact role of exogenous albumin in patients who undergo pancreatectomy remain controversial [7,8]. Researchers who oppose the use of exogenous albumin insisted that hypoalbuminemia is not a modifiable factor that can improve postoperative outcomes, but a result of poor prognosis after pancreatectomy [8].

In this study, we could not verify the positive effect of preemptive albumin administration on postoperative outcomes in patients undergoing pancreatectomy. Nevertheless, preemptive administration of albumin reduced the net-fluid balance during pancreatectomy. Despite the contribution of exogenous albumin during surgery, it did not lead to improved postoperative outcomes. We performed GDFT during pancreatectomy. In previous studies, GDFT helped optimize the volume status of patients without overuse of fluid and prevented acute edema that could interfere with surgery during PD [16]. The implementation of GDFT is also expected to lead to relatively low fluid volumes in both groups in this study compared with previous studies [1,2,16]. Although the efficacy of GDFT was not evaluated in this study, based on previous studies, we believe that the partial contribution of exogenous albumin to reducing the amount of fluid needed might be covered by an evidence-based fluid management strategy.

The serum albumin levels decreased after pancreatectomy compared to the preoperative values and did not recover until POD 5 in both groups, with the level more reduced in the control group. Although albumin replacement alleviated the decrease in serum albumin level in the albumin group, it did not change postoperative outcomes. The serum albumin level decreased after surgery for various reasons, including changes in albumin metabolism, extravascular loss, dilution due to fluid therapy, and injury to the EG layer [20]. The EG layer plays a significant role in intra- and extracellular liquid exchange and reabsorption of interstitial fluid [8,21], and is damaged by surgical trauma, the systemic stress response, and inflammatory reaction during the perioperative period [21]. Because the impaired EG layer cannot retain the infused albumin continuously, exogenous albumin temporarily raised the plasma oncotic pressure and reduced intraoperative net-fluid balance, but did not improve postoperative outcomes.

In the multiple logistic regression analysis, preoperative biliary drainage and a longer duration of surgery were risk factors for moderate postoperative complications, and these results were in line with those of previous studies. Povoski et al. [22] reported an association between preoperative biliary drainage and increased morbidity rates in patients who underwent PD. Maggino et al. [23] showed that a longer operative duration was independently associated with worse postoperative outcomes after pancreatic resection. Even after adjustment using a logistic regression model with preoperative biliary drainage, a longer duration of surgery remained a relevant risk factor for moderate postoperative complications.

The strength of our study was its prospective, randomized, and controlled trial design. Most previous studies that investigated the effect of exogenous albumin on postoperative outcomes used a retrospective or observational study design, in which inherently uncontrolled factors may have influenced the results. However, this study had several limitations. First, most participants showed a normal range of serum albumin levels before surgery in both groups. Although this trial focused on the empirical and preemptive use of albumin to prevent fluid overload and edema during anesthesia, the efficacy of exogenous albumin can be alleviated in patients with normal albumin levels. Second, various types of pancreatectomy were included. Although all surgeries involved pancreatic resections, the invasiveness or complexity of each surgery differed, which could affect postoperative surgical outcomes. After adjusting for the type of surgery, however, the results did not change in this study. Third, 13 patients in the control group underwent albumin replacement during the postoperative period because of significant hypoalbuminemia. Although surgeons were blinded to group allocation, and all patients were included in the analysis based on the intention-to-treat principle, these patients could influence the primary outcomes of the study.

## 5. Conclusions

Preemptive albumin infusion to prevent fluid overload and edema in patients undergoing pancreatectomy did not reduce the incidence of a Clavien–Dindo classification grade ≥ 2. Therefore, preemptive administration of exogenous human albumin to prevent edema during pancreatectomy is not recommended because of its lack of apparent benefit in improving postoperative outcomes.

## Figures and Tables

**Table 1:** Baseline characteristics of patient, operation, and anesthesia.

Variables	Control Group (*n* = 100)	Albumin Group (*n* = 100)	SMD
Patient Characteristics			
Age, years	64 ± 9	63 ± 10	0.086
Sex, male	56 (56)	60 (60)	0.081
Body mass index, kg/m^2^	23.6 ± 3.2	24.0 ± 3.0	0.128
ASA physical status ≥ III	8 (8)	6 (6)	0.078
Hypertension	37 (37)	40 (40)	0.062
Diabetes mellitus	32 (32)	23 (23)	0.203
Preoperative albumin, g/dL	4.2 (4.0–4.5)	4.2 (3.9–4.4)	0.183
Use of octreotide before surgery	46 (46)	43 (43)	0.060
Neo-adjuvant chemotherapy	10 (10)	6 (6)	0.148
Preoperative biliary drainage	30 (30)	45 (45)	0.314
Preoperative fistula risk score ^1^	3 (2–5)	3 (2–5)	0.036
Preoperative Braga score ^1^	3 (2–7)	3 (2–6)	0.080
Disease characteristics ^2^			
Pancreatic mass size, mm	20 (15–30)	24 (18–30)	0.107
Pancreatic mass texture, soft	20 (30)	24 (30)	0.007
Pancreatic duct size, mm	3 (2–4)	3 (2–4)	0.014
High-risk pathology ^3^	30 (31)	35 (36)	0.116
Portal vein invasion	8 (8)	10 (10)	0.074
TNM stage, I/II/III/IV	40/32/11/7	23/36/16/8	0.297
Operation			
Type of surgery			0.307
Pancreaticoduodenectomy ^4^	76 (76)	87 (87)	
RAMPS	23 (23)	11 (11)	
Total pancreatectomy	1 (1)	2 (2)	
Duration of surgery, min	318 ± 94	327 ± 71	0.101
Anesthesia			
Crystalloid infusion, mg/kg/h	3.7 (3.0–4.4)	3.0 (2.3–4.1)	0.447
Intraoperative blood loss, mL	250 (150–400)	300 (200–400)	0.013
Use of synthetic colloid	19 (19)	9 (9)	0.291
Intraoperative transfusion	5 (5)	4 (4)	0.048
Intraoperative fluid bolus ^5^	34 (34)	28 (28)	0.130
Cardiac index, l/min/m^2^	2.5 (2.2–3.1)	2.9 (2.4–3.4)	0.503
Use of vasopressor/inotrope	33 (33)	34 (34)	0.021

SMD: standardized mean difference; ASA: American Society of Anesthesiologists; TNM: tumor, node, and metastasis; RAMPS: radical antegrade modular pancreatosplenectomy. ^1^ Predictive risk scores were calculated in patients who underwent pancreaticoduodenectomy. The definition of each scoring system is presented in Table S1. ^2^ Total number of patients in each group was not 100 due to missing data. ^3^ High-risk pathology included pathologies other than pancreatic adenocarcinoma or pancreatitis. ^4^ Pancreaticoduodenectomy includes pylorus preserving or resecting pancreaticoduodenectomy and Whipple’s operation. ^5^ A 200 mL amount of crystalloid bolus was administered when stroke volume variation > 15%, except for when mean arterial pressure > 65 mmHg or urine output > 0.5 mL/kg/h.

**Table 2:** Postoperative complications and outcomes after pancreatectomy.

Variables	Control Group (*n* = 100)	Albumin Group (*n* = 100)	Risk Difference (%) (95% CI)	Effect Estimate ^1^ (95% CI)	*p* Value
Clavien–Dindo classification ≥ 2	38 (38)	47 (47)	9 (−5 to 23)	1.24 (0.89 to 1.71)	0.198
Clavien–Dindo grade 1/2/3/4/5	12/25/11/2/0	7/20/25/1/1			0.066
CR-POPF grade B,C	10 (10)	9 (9)	−1 (−9 to 7)	0.90 (0.38 to 2.12)	0.809
Delayed gastric emptying	9 (9)	16 (16)	7 (−2 to 16)	1.78 (0.82 to 3.83)	0.134
Pneumonia	1 (1)	3 (3)	2 (−2 to 6)	3.00 (0.32 to 28.35)	0.621
Surgical site infection	13 (13)	22 (22)	9 (−1 to 19)	1.69 (0.90 to 3.17)	0.094
Intra-abdominal abscess	7 (7)	9 (9)	2 (−6 to 10)	1.29 (0.50 to 3.32)	0.602
Postoperative hemorrhage	4 (4)	8 (8)	4 (−3 to 11)	2.00 (0.62 to 6.43)	0.373
Biliary fistula	2 (2)	0 (0)	−2 (−5 to 0)	N/R^2^	0.497
Biliary stricture	1 (1)	1 (1)	0 (−1 to 3)	1.00 (0.06 to 15.77)	>0.99
Acute kidney injury ^3^	2 (2)	3 (3)	1 (−3 to 5)	1.50 (0.26 to 8.79)	0.653
Postoperative transfusion	8 (8)	13 (13)			0.249
Postoperative albumin use	13 (13)	2 (2)			0.003

CI: confidence interval; CR-POPF: clinically-relevant postoperative pancreatic fistula. ^1^ Effect estimate is risk ratio (2-sided 95% CI) by Wald likelihood ratio approximation test and chi-square hypothesis tests. ^2^ Not reported (N/R) because of no patients in the albumin group. ^3^ Definition for acute kidney injury followed the Acute Kidney Injury Network classification.

**Table 3:** Multiple logistic regression for predictors of Clavien–Dindo classification grade ≥ 2.

Variables	Univariable			Multivariable		
	Odd Ratio	95% CI	*p* Value	Odd Ratio	95% CI	*p* Value
Intraoperative albumin infusion	1.45	0.82 to 2.54	0.199	1.61	0.76 to 3.40	0.215
Body mass index, per kg/m^2^	1.04	0.95 to 1.14	0.377			
Diabetes mellitus	0.78	0.41 to 1.48	0.447			
Preoperative albumin, per g/dL	0.44	0.21 to 0.92	0.030	0.64	0.29 to 1.41	0.268
Neo-adjuvant chemotherapy	0.59	0.20 to 1.77	0.347			
Preoperative biliary drainage	4.2	2.3 to 7.8	<0.001	3.63	1.94 to 6.80	<0.001
Type of surgery, PD	4.6	1.8 to 11.8	0.001	1.62	0.55 to 4.76	0.379
Duration of surgery, per min^1^	1.01	1.00 to 1.01	<0.001	1.01	1.00 to 1.01	0.002
Intraoperative crystalloid infusion, per mL/kg/h	1.19	0.96 to 1.02	0.108			
Use of synthetic colloid	1.20	0.54 to 2.69	0.651			
Intraoperative fluid bolus	1.05	0.83 to 1.32	0.678			
Cardiac index, per L/min/m^2^	0.78	0.52 to 1.17	0.231			

CI: confidence interval; PD: pancreaticoduodenectomy. Variables with standardized mean difference > 0.1 in the characteristics of patients, operations, and anesthesia are presented in Univariate column. ^1^ After adjusting for preoperative biliary drainage and type of surgery, a longer duration of surgery remained a relevant risk factor (per min; odds ratio: 1.01; 95% CI: 1.00 to 1.01; *p* = 0.015).

## Data Availability

The data presented in this study are available on request from the corresponding author.

## Supplementary Materials

The following supporting information can be downloaded at: https://www.mdpi.com/article/10.3390/jcm11030620/s1, Table S1: Risk assessment scoring systems to predict postoperative outcomes after pancreaticoduodenectomy; Table S2: Subgroup analysis of postoperative complications after pancreatectomy.
