# Supplementary material for: Preemptive Administration of Albumin during Pancreatectomy Does Not Reduce Postoperative Complications: A Prospective Randomized Controlled Trial
Source: J Clin Med. 2022 Jan 26;11(3):620. doi: 10.3390/jcm11030620 (PMC8837114; doi:10.3390/jcm11030620)
Supplement: Supplementary file 1 [file jcm-11-00620-s001.zip › jcm-1547344-supplementary.pdf]

Table S1. Risk assessment scoring systems to predict postoperative outcomes after pancreaticoduodenectomy

<table>
<tr><th>Scoring system</th><th colspan="3">Definition</th></tr>
<tr><td>Pancreatic fistula risk score</td><td colspan="3">A simple 10-point fistula risk score, accrued from International Study Group of Pancreatic Fistula risk factors, which accurately predicts the development of clinically relevant postoperative pancreatic fistula after pancreaticoduodenectomy.</td></tr>
<tr><td></td><td>Risk factor</td><td>Parameter</td><td>Points</td></tr>
<tr><td></td><td rowspan="2">Gland texture</td><td>Firm</td><td>0</td></tr>
<tr><td></td><td>Soft</td><td>2</td></tr>
<tr><td></td><td rowspan="2">Pathology</td><td>Pancreatic adenocarcinoma or pancreatitis</td><td>0</td></tr>
<tr><td></td><td>Ampullary, duodenal, cystic, islet cell</td><td>1</td></tr>
<tr><td></td><td rowspan="5">Pancreatic duct diameter, mm</td><td>≥ 5</td><td>0</td></tr>
<tr><td></td><td>4</td><td>1</td></tr>
<tr><td></td><td>3</td><td>2</td></tr>
<tr><td></td><td>2</td><td>3</td></tr>
<tr><td></td><td>≤ 1</td><td>4</td></tr>
<tr><td></td><td rowspan="4">Intraoperative blood loss, ml</td><td>≤ 400</td><td>0</td></tr>
<tr><td></td><td>401 - 700</td><td>1</td></tr>
<tr><td></td><td>701 - 1000</td><td>2</td></tr>
<tr><td></td><td>> 1000</td><td>3</td></tr>
</table>

Braga score	An 11-point prognostic score to predict postoperative outcome and major complications after pancreaticoduodenectomy.

Predictor	Categories	Scores
Pancreatic texture	Hard	0
	Soft	4
Pancreatic duct diameter, mm	> 3	0
	≤ 3	1
Operative blood loss, ml	< 700	0
	≥ 700	4
American Society of Anesthesiologist Score	I	0
	II	1
	III	2

Table S2. Subgroup analysis of postoperative complications after pancreatectomy

Claviden-Dindo classification ≥ 2	Control group (n = 100)	Albumin group (n = 100)	Risk difference (%) (95% CI)	Effect estimate[1] (95% CI)	P value
Subgroup					
Pancreaticoduodenectomy	33 (43)	45 (52)	8 (-7 to 24)	1.19 (0.86 to 1.65)	0.290
Other surgeries[2]	5 (21)	3 (23)	-2 (-30 to 26)	1.11 (0.31 to 3.91)	0.874

CI: confidence interval. [1]Effect estimate is risk ratio (2-sided 95% CI) by Wald likelihood ratio approximation test and Chi-square hypothesis tests. [2]Other surgeries included radical antegrade modular pancreatosplenectomy and total pancreatectomy.
